# Training and accreditation standards for pathologists undertaking clinical trial work

**DOI:** 10.1002/cjp2.124

**Published:** 2019-02-27

**Authors:** Gabrielle Rees, Manuel Salto‐Tellez, Jessica L Lee, Karin Oien, Clare Verrill, Alex Freeman, Ilaria Mirabile, Nicholas P West, on behalf of the National Cancer Research Institute (NCRI) Cellular‐Molecular Pathology (CM‐Path) clinical trials working group, Maggie Cheang, Manuel Rodriguez‐Justo, Emily Howlett, Laura Moretti, Maria Da Silva, Wong NACS, Sidonie Hartridge‐Lambert, Joseph M Beecham, Stephanie Traub, Sidath Katugampola, Sarah Blagden, James Morden, Max Robinson, Jacqueline James, Louise J Jones, Clare Craig, Philip Sloan, Gareth J Thomas, Philip Elliott, Owen J Driskell, Andy Hall

**Affiliations:** ^1^ Department of Cellular Pathology John Radcliffe Hospital Oxford UK; ^2^ Northern Ireland Molecular Pathology Laboratory Centre for Cancer Research and Cell Biology, Queens University Belfast UK; ^3^ Strategy and Initiatives National Cancer Research Institute London UK; ^4^ Institute of Cancer Sciences – Pathology University of Glasgow Glasgow UK; ^5^ Nuffield Department of Surgical Sciences University of Oxford, and Oxford NIHR Biomedical Research Centre Oxford UK; ^6^ Department of Pathology University College London Hospitals NHS Foundation Trust London UK; ^7^ ECMC Programme Office, Experimental Cancer Medicine Centres (ECMCs) Network London UK; ^8^ Pathology and Data Analytics Leeds Institute of Medical Research at St. James's, University of Leeds Leeds UK; ^9^ Institute of Cancer Research Clinical Trials and Statistics Unit, The Institute of Cancer Research 15 Cotswold Road, Surrey SM2 5NG UK; ^10^ Department of Gastroenterology University College Hospitals London London UK; ^11^ Precision Medicine Team, Cancer Research UK London UK; ^12^ Institute of Cancer Research Clinical Trials and Statistics Unit, The Institute of Cancer Research 15 Cotswold Road, Surrey SM2 5NG UK; ^13^ Sectra UK; ^14^ Department of Cellular Pathology Southmead Hospital Bristol; ^15^ Bristol‐Myers Squibb London UK; ^16^ Nanostring Washington USA; ^17^ Centre for Drug Development, Cancer Research UK London UK; ^18^ Centre for Drug Development, Cancer Research UK London UK; ^19^ Department of Oncology University of Oxford Oxford UK; ^20^ Institute of Cancer Research Clinical Trials and Statistics Unit, The Institute of Cancer Research 15 Cotswold Road, Surrey SM2 5NG UK; ^21^ Centre for Oral Health Research, Newcastle University Newcastle upon Tyne NE2 4BW; ^22^ School of Medicine, Dentistry and Biomedical Sciences, Centre for Cancer Research and Cell Biology, Institute for Health Sciences Queens University Belfast; ^23^ Centre for Tumour Biology, Barts Cancer Institute, Barts and the London School of Medicine and Dentistry London UK; ^24^ Genomics England London UK; ^25^ Department of Cellular Pathology Newcastle upon Tyne Hospitals NHS Trust Newcastle upon Tyne UK; ^26^ Faculty of Medicine Cancer Sciences Unit Southampton University Somers Building, Southampton UK; ^27^ Centre for Tumour Biology, Barts Cancer Institute, Barts and the London School of Medicine and Dentistry London UK; ^28^ Department of Clinical Biochemistry University Hospitals of North Midlands Stoke‐on‐Trent Staffordshire UK; ^29^ Institute for Applied Clinical Sciences, University of Keele Stokeon‐ Trent Staffordshire UK; ^30^ National Institute for Health Research Clinical Research Network West Midlands.; ^31^ Newcastle University Newcastle upon Tyne NE2 4BW

**Keywords:** clinical trials, training and accreditation, NCRI CM‐Path

## Abstract

Clinical trials rely on multidisciplinary teams for successful delivery. Pathologists should be involved in clinical trial design from the outset to ensure that protocols are optimised to deliver maximum data collection and translational research opportunities. Clinical trials must be performed according to the principles of Good Clinical Practice (GCP) and the trial sponsor has an obligation to ensure that all of the personnel involved in the trial have undergone training relevant to their role. Pathologists who are involved in the delivery of clinical trials are often required to undergo formal GCP training and may additionally undergo Good Clinical Laboratory Practice training if they are involved in the laboratory analysis of trials samples. Further training can be provided via trial‐specific investigator meetings, which may be either multidisciplinary or discipline‐specific events. Pathologists should also ensure that they undertake External Quality Assurance schemes relevant to the area of diagnostic practice required in the trial. The level of engagement of pathologists in academia and clinical trials research has declined in the United Kingdom over recent years. This paper recommends the optimal training and accreditation for pathologists undertaking clinical trials activities with the aim of facilitating increased engagement. Clinical trials training should ideally be provided to all pathologists through centrally organised educational events, with additional training provided to pathologists in training through local postgraduate teaching. Pathologists in training should also be strongly encouraged to undertake GCP training. It is hoped that these recommendations will increase the number of pathologists who take part in clinical trials research in order to ensure a high level and standard of data collection and to maximise the translational research opportunities.

## Introduction

Pathologists play an important role in the design and delivery of clinical trials internationally, whether these are to test the effectiveness of new devices or novel treatments e.g. drugs or surgical procedures. Clinical trial pathologists should be involved in trial design from the outset to ensure that the collection of tissue and/or morphological and molecular data for biomarker analysis/translational research is performed to a consistently high standard 1. All too often clinical trials are designed without the involvement of pathologists leading to incomplete data collection, unworkable protocols and lost translational research opportunities. In our opinion, there is an urgent need to increase the number of pathologists involved in clinical trial delivery, clinical trial design (through membership of trial management groups) and clinical trial monitoring (through membership of data monitoring committees in order to improve the overall quality of clinical trials research). We believe that pathologists should be involved in clinical trials planning to some extent in almost all trials to optimise the quality of pathological endpoint generation and ensure that translational opportunities are maximised.

Whilst it is recognised that there is *ad hoc* training available for clinical trials pathologists from a variety of sources, there is limited existing guidance as to the minimum amount of training that is essential, or indeed desirable, for them to undertake. This guidance is not currently available from one unifying source. Current sources of training often fail to take into account the required level of engagement in a clinical trial. Pathologist involvement in clinical trials should be seen as a mainstream clinical activity and not restricted to pathologists with formal academic appointments. Pathologists will have a variable input into the running and management of clinical trials, with most pathologists providing ‘local’ input in their centre e.g. collection of tissue, completion of pathology case report forms etc. A more limited number of pathologists will have more of a ‘central’ role including chief investigator/central pathology lead with responsibility for generating the protocol and design of the pathological endpoints.

Over recent years there has been a significant decline in academic pathologists in the United Kingdom (UK) resulting in a low level of engagement in clinical trials work 2. In 2016 the National Cancer Research Institute (NCRI) launched the Cellular Molecular Pathology (CM‐Path) initiative to address this and a number of other issues in order to increase engagement and capacity in both academic and molecular pathology 3. The Clinical Trials workstream of CM‐Path organised a 1 day workshop in March 2017, one of the objectives of which was to determine the optimal training and accreditation for pathologists taking part in clinical trials work within the UK, bringing in expertise from all CM‐Path workstreams. Other topics considered during the workshop included regulation and accreditation for laboratories taking part in clinical trials, and optimal scoring and reporting of clinical trial specimens to include digital pathology and image analysis, which has been summarised in a separate manuscript 4. It was hoped that dissemination of this information would provide pathologists with the knowledge to be able to get more involved in clinical trials work, either as a local pathologist contributing to the collection of data, or as a central pathologist with management responsibilities including protocol design. The workshop formulated ‘practice points’ to help pathologists navigate clinical trials training and accreditation and these are placed in italics at the end of each section and compiled in Table 1.

**Table 1:** Summary of clinical trials training and accreditation practice points

Basic GCP training, as a minimum, should be strongly encouraged for all clinical staff who are involved in any aspect of clinical trial work. The specific type of GCP training undertaken should be aligned to cover the specific tasks that the researcher is expected to perform. As a minimum all pathologists should understand the basics of clinical trial governance. Additional training should be considered for pathologists undertaking translational research in a central laboratory e.g. GCLP training.
In the UK, CM‐Path should engage with the NIHR CRN to develop an online GCP training resource specifically tailored to pathologists and translational researchers involved in clinical trials research.
GCLP training should be considered as an additional training resource for all pathologists and laboratory staff who undertake translational laboratory analysis of clinical trial samples.
Trial‐specific investigator meetings are an invaluable source of information and training and are highly recommended during the set‐up of a trial.
Pathologists should participate in specialist EQA schemes appropriate to the field of their clinical research involvement.
Pathologists in training should be strongly encouraged to learn about the role of pathologists in clinical trials and undertake further training in this area where there is interest e.g. GCP training.
All pathologists should have access to basic training in the main concepts involved in clinical trials research through national workshops.

In the UK, pathologist engagement can be increased by ensuring that all of the NCRI clinical studies groups have pathologist representation to facilitate review of at least the national portfolio trials, encouraging new generations of early career pathologists to be engaged with clinical trials research early in their careers, and ensuring that pathologists have access to funding to ‘buy out’ their time for research (a key objective of the CM‐Path clinical trials workstream). Further review of all trials by the CM‐Path Clinical Trials Pathology Advisory Group (CT‐PAG) prior to funding applications is also to be encouraged.

The aim of the workshop was to evaluate the existing training and accreditation guidance, along with current sources of training, and to produce a consensus statement that could act as a single source of guidance for clinical trials pathologists, as well as directing them to potential sources of further information. It was recognised that there is an urgent need for such guidance, as part of a move to assuring high quality pathology in clinical trials, and to maximise the available translational research opportunities. The working group appreciated the importance of achieving a balance between assuring high quality pathology in clinical trials and not imposing unrealistic demands that might deter potential clinical trials pathologists from taking part.

## Overview of current guidance

The Medicines and Healthcare Products Regulatory Agency (MHRA) guidance on Good Clinical Practice (GCP) quotes the Clinical Trials Regulations which state that ‘No person shall conduct a clinical trial other than in accordance with the conditions and principles of good clinical practice’ and thus advises that all personnel involved in the analysis and evaluation of clinical trial samples should receive GCP training ‘commensurate with their roles and responsibilities.’ Although there is no legal requirement to undergo formal GCP training, such training is often considered to be mandatory by clinical trials sponsors, who will usually ask all personnel involved in the trial (including named site pathologists) to provide evidence of completion of training. This training should ordinarily be refreshed every 2 years. Other pathologists who contribute to the delivery of the trial e.g. by reporting cases from enrolled patients, are not usually mandated by sponsors to undertake GCP training as they are technically acting under the guidance of the named site pathologist. If the named site pathologist will not personally deliver all of the trial‐related pathology in the centre, it is important that they still retain oversight and communicate clearly the requirements of the trial to the rest of their team. Laboratory staff who are involved in the analysis of clinical trial samples are often not expected to undertake GCP training, although they may alternatively undertake Good Clinical Laboratory Practice (GCLP) training to ensure that they can perform their role according to the principles of GCP. Ultimately, the type of training required and who should undergo training is determined by the clinical trial sponsor, who retains overall responsibility for the delivery of the trial.

### GCP training

GCP is an internationally agreed set of standards that govern how clinical research is conducted to ensure that the rights and safety of the participants are protected and that the research data are reliable. GCP training is readily available and can be undertaken either as a face‐to‐face exercise or via on‐line training, both of which should be made available free of charge for clinical staff involved in clinical trials work. Some organisations offer their own in‐house training programmes. As an alternative, free on‐line training is easy to undertake at a convenient time and can be accessed via the National Institute for Health Research (NIHR) Clinical Research Network (CRN) 5. There are a number of different NIHR CRN courses available depending on the type and level of training required, although currently none is specifically tailored to pathologists or translational researchers. This could be addressed by CM‐Path working with the NIHR CRN to develop a set of appropriate resources. It is important that any training undertaken covers the specific tasks the researcher will be expected to perform. On‐line GCP training is not an onerous undertaking and can usually be completed within approximately 3 h. The training usually covers all of the important aspects of clinical trials working including the regulations for setting up and delivering clinical trials, the roles and responsibilities of organisations and individuals involved, required documentation, consenting and safety.

*Basic GCP training, as a minimum, should be strongly encouraged for all clinical staff who are involved in any aspect of clinical trial work. The specific type of GCP training undertaken should be aligned to cover the specific tasks that the researcher is expected to perform. As a minimum all pathologists should understand the basics of clinical trial governance. Additional training should be considered for pathologists undertaking translational research in a central laboratory e.g. GCLP training (see below)*.

*In the UK, CM‐Path should engage with the NIHR CRN to develop an online GCP training resource specifically tailored to pathologists and translational researchers involved in clinical trials research*.

### GCLP training

GCLP training essentially covers the regulation of the laboratory analysis of clinical trials samples according to the principles of GCP. Whilst GCLP standards are comprehensive, they are not a recognised legal framework unlike GCP. Despite this, GCLP training is potentially very useful for laboratory staff involved in clinical trial work and should be considered as an additional training resource for pathologists who undertake translational laboratory analysis of clinical trial samples. Again this can be provided by on‐line training 6, although this is not free of charge as far as we are aware unlike the NIHR CRN GCP courses.

*GCLP training should be considered as an additional training resource for all pathologists and laboratory staff who undertake translational laboratory analysis of clinical trial samples*.

## Additional training and quality assurance available

### Trial‐specific investigator meetings

Many clinical trials will hold trial‐specific investigator meetings, particularly at the start of a trial and possibly at regular intervals during it. These meetings are an opportunity for the trial management team to disseminate the plans for the conduct of the trial, and are a very useful source of trial specific information for clinical trials pathologists. They are also an opportunity for pathologists to ask questions to clarify anything that may not be clear in the protocol. Pathologists should be recognised as crucial to the successful delivery of many clinical trials and essential to maximise the translational research opportunities and should be actively encouraged to attend by the wider multidisciplinary team. There are two main types of investigator meeting:

1. Multidisciplinary investigator meetings – for all trial investigators to attend.
2. Discipline‐specific workshops – where investigators from a single discipline attend to discuss the discipline‐specific requirements of the trial.

Both of these types of meeting are valuable for clinical trials pathologists to attend, but pathology‐specific workshops are especially useful if pathologists are involved in primary or secondary endpoint analysis, particularly if these are obtained by non‐routine methods. Such workshops may contain a practical discipline‐specific training element, particularly if the data to be collected are non‐routine. Those of us with personal experience (NPW) have found that pathology‐specific workshops are invaluable, both as a source of information and as a potential venue for discussing issues and practical difficulties with ongoing trial involvement. They are a useful forum for a two‐way dialogue between the central pathologists who have written the protocol and the site pathologists who carry out the trial to agree on the deliverability of the protocol for pathology end point generation. Indeed it is not unusual for protocols to be modified on the basis of feedback from local pathologists who will perform the day‐to‐day data collection.

Whilst face‐to‐face meetings are ideal, funding needs to be obtained by the sponsor to meet the running costs and not all pathologists are likely to be able to attend a single event meaning that multiple workshops may be required. Meetings can satisfactorily be alternatively run as a web‐based session (keeping time and cost demands to a minimum) and/or can be attended by one representative pathologist from each site (who can then distribute the information to other pathologists in their local team), and this should be covered by the pathology support funding for the trial.

An additional benefit of attendance at trial‐specific investigator meetings is that Continuing Professional Development (CPD) points are often allocated for attendance, thus providing an additional incentive for pathologists to attend.

*Trial‐specific investigator meetings are an invaluable source of information and training and are highly recommended during the set‐up of a trial*.

### Specialist EQA schemes

It is important that clinical trials pathologists participate in an External Quality Assurance (EQA) scheme relevant to the field of their clinical research involvement e.g. a pathologist involved in colorectal cancer trials would be expected to subscribe to the national gastrointestinal EQA scheme. This helps to ensure a high level of quality in that diagnostic field, as well as providing demonstrable competence in the form of a certificate of participation for MHRA inspections. However, it is recognised that for some specialist areas of work, appropriate EQA schemes do not exist or are oversubscribed. In trials where molecular testing is performed within pathology laboratories, it is important that laboratories subscribe to the relevant molecular EQA schemes where these are available.

*Pathologists should participate in specialist EQA schemes appropriate to the field of their clinical research involvement*.

## Training for the future

Any pathologist with an interest in clinical trials should be familiar with the generic governance of clinical trials as well as concepts such as biobanking and biomarker validation etc according to the requirements of the trial(s) they are involved in. Specific items of governance that are often poorly understood by pathologists include the limitations of contracts, particularly in commercial trials, that can impact on the ability to publish the result. Specific training in clinical trial contract issues e.g. non‐disclosure, intellectual property etc would be very useful to understand potential conflicts of interest at an early stage of trial design.

Whilst a central clinical trial pathologist might be expected to have in‐depth knowledge of the whole of the trial process, it is probably not necessary or feasible for every collaborating local trial pathologist to demonstrate such expertise. In general, central trial pathologists who sit on the trial management group and are involved in writing the protocol/analysis should be expected to have a greater level of training; however, currently, there is no formal platform in which training in these areas can easily be delivered. However, we believe that all pathologists should have a basic knowledge of clinical trials research given the large number of clinical trials being delivered across the health service including in many small district general hospitals. Various concepts for the provision of basic training necessary for clinical trials pathologists have been considered.

### Involving pathologists in training

The UK Royal College of Pathologists (RCPath) Histopathology curriculum requires that all pathologists in training have the appropriate knowledge, skills and behaviours to undertake research on completion of training 7. There is no specific mention of training in clinical trials research. The working group agreed that an element of basic clinical trials training should be provided within this framework, and would be best achieved by integration into local Fellowship of the Royal College of Pathologists (FRCPath) training programmes. In addition, specific training modules could be developed nationally through CM‐Path for clinical trials pathologists. It was also agreed that the RCPath curriculum could specifically state that pathologists in training should have a basic knowledge of the concepts surrounding pathology involvement in clinical trials. CM‐Path will continue to work with the RCPath to ensure that this is recognised.

One other source of training for pathologists in training that could easily be provided without additional funding would be the requirement to undertake GCP training in stage B of FRCPath training. Potential benefits of providing basic clinical trials training to pathologists in training include:

- Early exposure of pathologists to clinical trial work, which could help to address the current shortage of pathologists willing to get involved in the development and delivery of trials.
- Increased awareness of the benefits of clinical trials, both for patients and for the future development of pathology.
- Opening up possibilities for the negotiation of clinical trials involvement as part of new consultant/staff pathologist job plans.
- Ensuring that pathologists in training meet the requirements of the current Histopathology curriculum in terms of the expected knowledge, skills and behaviours around research.

It was recognised that incorporating compulsory GCP training into an already packed training programme might be unrealistic; however, it is not currently clear whether specific research training is provided across all training programmes in the UK and where it is provided, how this is delivered. As a minimum, the working group felt that pathologists in training should be strongly encouraged to undertake GCP training, directing pathologists in training to suitable, easily accessible, free training modalities e.g. on‐line training available through the NIHR CRN. Teaching the basic concepts of research should already be delivered to meet the curriculum requirements, and training programmes should be encouraged to specifically cover the basics of clinical trials work during their regional teaching programmes or as part of a centrally organised teaching event. In addition it may be possible to work with medical schools to deliver mandatory GCP training during undergraduate courses so that medical graduates have a clear understanding of the roles and responsibilities of clinicians involved in clinical trials research.

*Pathologists in training should be strongly encouraged to learn about the role of pathologists in clinical trials and undertake further training in this area where there is interest e.g. GCP training. CM‐Path will continue to work with the RCPath to ensure that the curriculum recognises the importance of training in the principles of clinical trials research*.

It is important that pathologists in training recognise the collective and individual value of taking part in clinical trials research. Beyond the benefits in terms of the advancement of medical knowledge, pathologists who engage in clinical trials activity will find several opportunities to improve their curriculum vitae through peer reviewed publications (often in high impact factor journals), attendance at investigator meetings, a deeper understanding of clinical trials design/governance and learning new specialist areas of pathology. With commercial trials activity, there is also the opportunity to obtain consultancy fees either for personal gain or to contribute towards departmental research funds.

Whilst training in the optimal collection of tissue/data and clinical trial governance is important, it is also critical that all pathologists in training are trained as competent ‘morpho‐molecular’ diagnostic pathologists 8, 9, in order to support the stratified allocation of patients into national prospective randomised clinical trials e.g. MRC FOCUS4 10 and the CRUK National Lung Matrix Trial 11. It is also of fundamental importance that we train a significant subset of pathologists as effective clinical/translational researchers to play a major role in the design of new trials and undertake retrospective exploratory and validatory biomarker analyses on clinical trial samples.

Senior pathologists engaged in clinical trials research should be strongly encouraged to consider succession planning by engaging junior consultant/staff pathologist colleagues or pathologists in training as deputies in order to provide ‘on the job’ training. They should be recognised as formal deputies on the trial delegation log and must ensure that the basic training requirements have been met i.e. GCP training.

### Central workshops for training

It was recognised that whilst it is relatively easy to provide clinical trial training to pathologists in training through FRCPath teaching events and changes to the curriculum, it is more difficult to provide training to established consultants/staff pathologists. One potential solution is the organisation of central workshops, which would be open to both pathologists in training and current consultants/staff pathologists and would attract CPD points for participation. To date CM‐Path has already held two such events to stimulate interest and involvement in clinical trials research from pathologists at all stages of their career.

*All pathologists should have access to basic training in the main concepts involved in clinical trials research through national workshops*.

A summary of potential training opportunities is provided in Table 2.

**Table 2:** Summary of the clinical trials training opportunities for pathologists discussed at the CM‐Path workshop with guidance on when to consider the various activities

Training activity	When should this training activity be considered?
Good Clinical Practice (GCP) training	All pathologists who undertake clinical trials work should be strongly encouraged to undertake GCP training as a minimum – clinical trial sponsors usually insist on collecting documentary evidence of GCP training for pathologists who are listed on the site delegation log. Pathologists in training should consider undertaking GCP training at an early stage to understand the basics of clinical trials governance. Local training programmes could consider recommending this – at an appropriate stage of training and pathologists who are actively engaged in clinical trials should recommend this training to their junior staff.
Good Clinical Laboratory Practice (GCLP) training	GCLP training covers the elements of GCP that are relevant to laboratory practice. This should be considered in addition to basic GCP training for pathologists who are involved in laboratory translational research and also should be undertaken by laboratory scientists and technicians dealing with clinical trials samples.
Multidisciplinary trial investigator meetings	Helpful for the lead pathologist from each site to attend to understand the wider context of the trial and maximise translational research opportunities. These meetings may be used to disseminate the results at the end of the trial. The lead pathologist can feed back to local colleagues who will assist in trial delivery.
Pathology specific trial investigator meetings	Very useful to discuss the practical issues of protocol delivery, especially the deliverability of pathological endpoints.
Specialist EQA schemes	Where relevant specialist clinical reporting schemes exist, pathologists should subscribe to schemes relevant to the clinical area of interest. Where molecular testing forms part of the trial, the relevant EQA schemes should be undertaken where these are available (https://www.ukneqas‐molgen.org.uk/).
FRCPath training programmes	Postgraduate pathology training programmes should consider inviting pathologists engaged in clinical trials activity to present the basics of clinical trials research and give some examples of how best to get involved.
Deputising for senior clinical trials pathologists	Pathologists in training or consultants/staff pathologists with no/limited trials experience should consider deputising for senior clinical trials pathologists to begin to develop some experience within a supportive environment. This will assist senior trials pathologists with succession planning.
Central workshops	Pathologists in training and consultant/staff pathologists should consider attending central workshops e.g. those organised by CM‐Path, covering the basics of clinical trials research with tips on how to get involved.

## Conclusion

Pathologists are vital to the successful delivery of clinical trials, and should be involved in the design and delivery of all trials to ensure maximum data collection and translational opportunities. There are few mandatory training requirements beyond GCP training, but other sources of education include GCLP training, trial‐specific investigator meetings, specialist EQA schemes, dedicated sessions during local postgraduate teaching, and centrally organised events. In the UK, CM‐Path will continue to work with the RCPath to ensure that the curriculum recognises the importance of training in clinical trials research to ensure that we create a generation of pathologists with an interest in and the skills required to deliver successful clinical trials with quality assured pathology support and maximal translational research opportunities.

## Author contributions statement

CV conceived and chaired the NCRI CM‐Path Quality Assurance in Clinical Trials Workshop. All authors contributed to the workshop. GR and NW composed the first draft of the manuscript. All authors critically reviewed and approved the final version of the manuscript.
